# The absence of the autoimmune regulator gene (*AIRE*) impairs the three-dimensional structure of medullary thymic epithelial cell spheroids

**DOI:** 10.1186/s12860-022-00414-9

**Published:** 2022-03-24

**Authors:** Ana Carolina Monteleone-Cassiano, Janaina A. Dernowsek, Romario S. Mascarenhas, Amanda Freire Assis, Dimitrius Pitol, Natalia Chermont Santos Moreira, Elza Tiemi Sakamoto-Hojo, João Paulo Mardegan Issa, Eduardo A. Donadi, Geraldo Aleixo Passos

**Affiliations:** 1 Program of Basic and Applied Immunology, Ribeirão Preto Medical School, University of São Paulo, Ribeirão Preto, SP Brazil; 2 Molecular Immunogenetics Group, Department of Genetics, Ribeirão Preto Medical School, University of São Paulo, Ribeirão Preto, SP 14049-900 Brazil; 3 Institute for Energy and Nuclear Research, University of São Paulo, São Paulo, SP Brazil; 4 Department of Basic and Oral Biology, School of Dentistry of Ribeirão Preto, University of São Paulo, Ribeirão Preto, SP Brazil; 5 Department of Genetics, Ribeirão Preto Medical School, University of São Paulo, Ribeirão Preto, SP Brazil; 6 Department of Biology, Faculty of Philosophy, Sciences and Letters at Ribeirão Preto, University of São Paulo, Ribeirão Preto, SP Brazil; 7 Division of Clinical Immunology, Department of Medicine, Ribeirão Preto Medical School, University of São Paulo, Ribeirão Preto, SP Brazil; 8 Center for Cell-Based Therapy in Dentistry, School of Dentistry of Ribeirão Preto, University of São Paulo, Ribeirão Preto, SP Brazil; 9 Laboratory of Genetics and Molecular Biology, Department of Basic and Oral Biology, School of Dentistry of Ribeirão Preto, University of São Paulo, Ribeirão Preto, SP Brazil

**Keywords:** *AIRE* gene, Medullary thymic epithelial cells, mTECs, Cell adhesion, Spheroid, 3D cell culture

## Abstract

**Background:**

Besides controlling the expression of peripheral tissue antigens, the autoimmune regulator (*AIRE*) gene also regulates the expression of adhesion genes in medullary thymic epithelial cells (mTECs), an essential process for mTEC-thymocyte interaction for triggering the negative selection in the thymus. For these processes to occur, it is necessary that the medulla compartment forms an adequate three-dimensional (3D) architecture, preserving the thymic medulla. Previous studies have shown that *AIRE* knockout (KO) mice have a small and disorganized thymic medulla; however, whether *AIRE* influences the mTEC-mTEC interaction in the maintenance of the 3D structure has been little explored. Considering that *AIRE* controls cell adhesion genes, we hypothesized that this gene affects 3D mTEC-mTEC interaction. To test this, we constructed an in vitro model system for mTEC spheroid formation, in which cells adhere to each other, establishing a 3D structure.

**Results:**

The comparisons between *AIRE* wild type (*AIRE*^*WT*^) and *AIRE* KO (*AIRE*^*−/−*^) 3D mTEC spheroid formation showed that the absence of *AIRE:* i) disorganizes the 3D structure of mTEC spheroids, ii) increases the proportion of cells at the G0/G1 phase of the cell cycle, iii) increases the rate of mTEC apoptosis, iv) decreases the strength of mTEC-mTEC adhesion, v) promotes a differential regulation of mTEC classical surface markers, and vi) modulates genes encoding adhesion and other molecules.

**Conclusions:**

Overall, the results show that *AIRE* influences the 3D structuring of mTECs when these cells begin the spheroid formation through controlling cell adhesion genes.

## Background

The three-dimensional (3D) structure of the thymus is composed by two histologically distinct but closely related compartments. The major component of the thymic stromal cells is epithelial cells, which generate the 3D organized meshwork structure. The cortex is mainly formed by cortical thymic epithelial cells (cTECs), exhibiting the CD45^−^, EpCam^+^, Ly51^+^, UEA1^−^ phenotype, and the medulla is primarily formed by medullary thymic epithelial cells (mTECs), which in mice present the CD45^−^, EpCam^+^, Ly51^−^, UEA1^+^ phenotype [1–3]. Other cell types include thymic seeding progenitors, thymocytes at different stages of maturation, including double-negative (CD4^−^CD8^−^), double-positive (CD4^+^CD8^+^), and single-positive (CD4^+^CD8^−^ and CD4^−^CD8^+^) thymocytes, thymic B cells, macrophages, dendritic cells, Hassall’s corpuscles, and mesenchymal thymic cells, which are required for epithelial cell proliferation, composing the unique 3D structure of the organ [2].

The thymic 3D architecture offers a particular microenvironment for the positive and negative selection of developing thymocytes [4–6]. The thymic cortex is implicated in the positive selection of thymocytes that express functional T-cell receptors (α/β TCRs). The medulla is involved in the negative selection of autoreactive thymocytes, which avidly recognize self-peripheral tissue antigens (PTAs) presented on the surface of mTECs. This unique pattern of gene expression allows mTECs to express more than 19,000 protein-encoding genes, including the “ectopic” genes that encode PTAs, a phenomenon known as promiscuous gene expression (PGE) [4, 7–10]. At present, no other cell type is known that expresses such a large set of genes [11–13]. The positive and negative selection processes are crucial for the induction of central immune tolerance that prevents aggressive autoimmunity [9, 14].

The autoimmune regulator gene (*AIRE)* is the primary transcriptional regulator of PTAs in mTECs*,* whose encoded Aire protein unleashes stalled RNA Pol II in the chromatin to proceed with the elongation phase of the transcription [15, 9, 10]. A second controller of PGE in mTECs is the forebrain embryonic zinc finger-like protein 2 (*Fezf2*) that plays a role as a classical transcription factor and thus binds directly to DNA in specific promoter regions [4, 16].

Given the non-specificity of RNA Pol II in transcribing genes and given that the *Aire* protein in mTECs unleashes this enzyme on chromatin, *AIRE* ends up controlling the expression of a diverse range of mRNAs. In addition to PTAs, *AIRE* also regulates the expression of genes involved in cell adhesion, such as the extracellular matrix (ECM) constituent Lama1, the CAM family adhesion molecules Vcam1 and Icam4, which control mTEC-thymocyte adhesion [17, 18]. Cell adhesion corresponds to an essential biological process in the structure and function of the thymus. The adequate adhesion of TECs increases the efficiency for T-cell development [19, 20]. Besides adhesion molecules, the formation of a dense cellular network is necessary for the maturation of thymocytes, which is composed of ECM proteins, such as laminins, integrins, collagens, and fibronectins, as well as soluble molecules such as hormones, cytokines, chemokines, and growth factors that are also mediated by *AIRE* [21].

*AIRE* ^*−/−*^ mice have small and disorganized medulla of the thymus [22], and little is known about whether Aire influences the mTEC-mTEC adhesion in a 3D thymus structure. Two experimental strategies enable the study of the in vitro formation of the thymus’s 3D structure that reproduces its microenvironment with the extracellular matrix and thymic epithelial cells. One of these strategies is the thymus re-aggregation [19, 20], and the other is the 3D organotypic culture [23]. In the re-aggregation model, the thymus tissue is devoid of cells (decellularized), but retains most of the microenvironment of the extracellular matrix that could support the re-aggregation between TECs. When transplanted into athymic nude mice, the re-aggregate thymus organoids can receive lymphocyte progenitors derived from bone marrow and develop a diverse and functional T-cell repertoire [19, 20].

In the search for an experimental model that could mimic the thymic microenvironment closest to that found in vivo, Pinto et al. [23] adapted a 3D organotypic co-culture, preserving the main characteristics of mTECs, such as proliferation and differentiation. This strategy helped to identify molecular components and pathways involved in the mTEC differentiation and promiscuous gene expression. Other studies used stem cells to form “thymospheres,” permitting the study of the factors necessary for thymocyte development [24, 25]. Recently, a 3D culture substrate has been reported that allowed TECs to survive and proliferate, using electrospun fibrous meshes (eFMs) functionalized with fibronectin. The mTECs presented increased proliferation, viability, and protein synthesis when cultured on fibronectin-functionalized eFMs (FN-eFMs) [26].

In the present study, we asked whether *AIRE* regulates the adhesion between mTECs during the in vitro formation of a 3D structure. For this, we developed a model system in which mTECs are grown in non-adherent agarose micro-wells and adhere to form spheroids. The comparison between *AIRE* wild type (*AIRE*^*WT*^) versus *AIRE*^*−/−*^ mTECs allowed us to evaluate the influence of *AIRE* in the adhesion between these cells during the spheroid formation.

## Results

### The absence of *AIRE* disorganizes the initial phases of spheroid formation

**Fig. 1:**
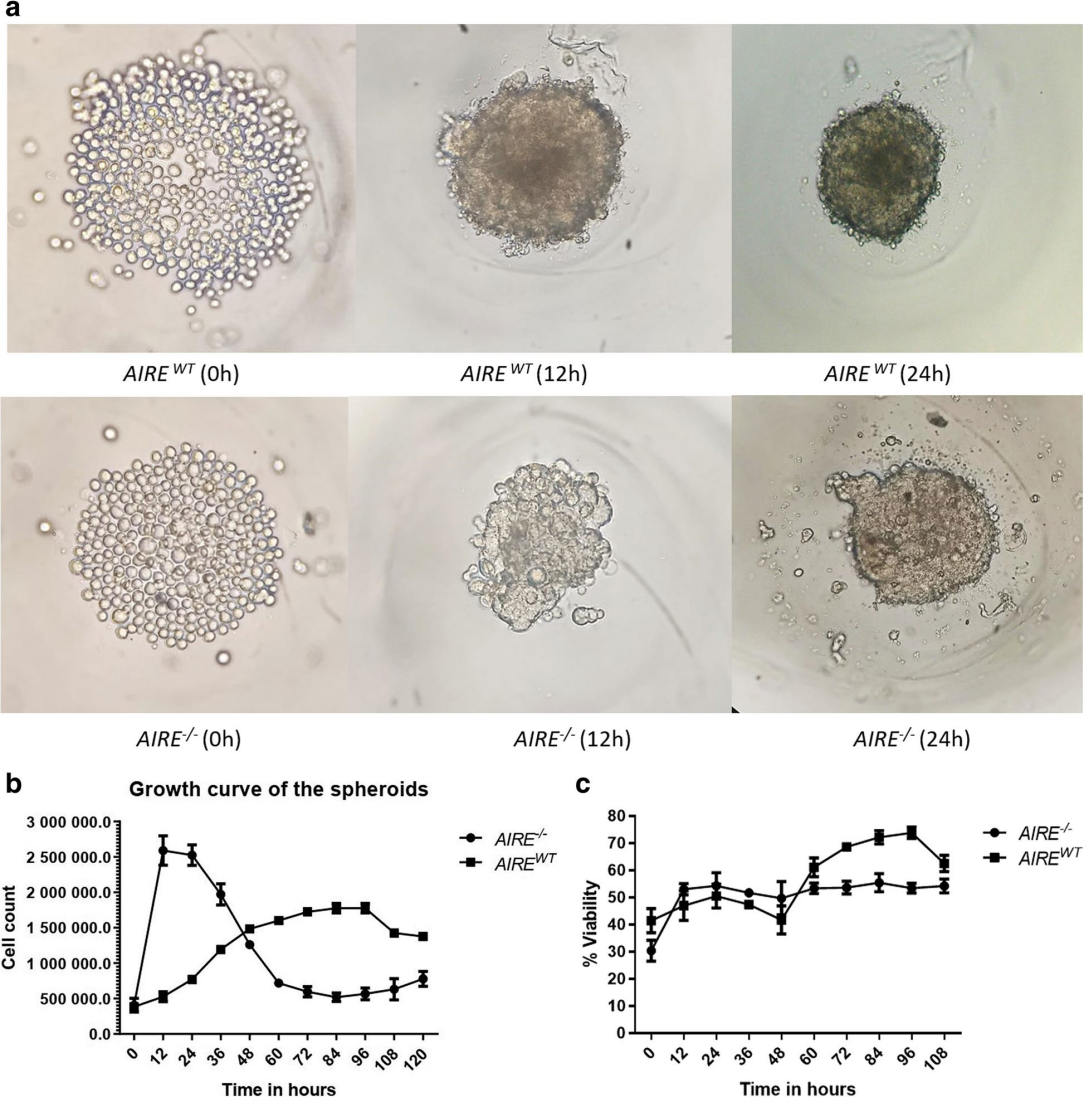
Comparisons between *AIRE*^*WT*^ and *AIRE*^*−/−*^ during the spheroid formation process. **A** Inverted light microscopy (40 x magnification) showing the spheroid formation in agarose micromolds starting from the deposition of 1 × 10^5^ mTECs, **B** Spheroid growth curves from 0 to 108 h, time-point values correspond to the mean and ± SD from three independent replicates. **C** Spheroid cell viability from 0 to 108 h of growth. One representative experiment of *n* > 3 is shown

Figure 1A shows the beginning of spheroid growth (0 h), comparing *AIRE*^*WT*^ mTEC 3.10 vs. *AIRE*^*−/−*^ mTEC 3.10E6. The cultures started from the seeding of mTEC cells (0 h) until the complete 3D spheroid formation (24 h). A compact real-time video of the dynamics of the spheroid formation, recorded from 0 to 24 h, is shown in the supplemental material video 1 (available at www.rge.fmrp.usp.br/passos/esferoides). The results show that the mTEC cell lines (*AIRE*^*WT*^ or *AIRE*^*−/−*^) provide morphologic differences during the development of spheroids. The *AIRE*^*WT*^ spheroids consolidate and are firmly compact at 12 h of culture, whereas the *AIRE*^*−/−*^ spheroids showed a delay in cell-cell adhesion and consolidate only after 20 h of culture. This finding indicates that in the absence of *AIRE*, mTEC-mTEC adhesion is impaired and the spheroid formation is delayed.

To better characterize the 3D spheroid model system, we constructed a growth curve counting cells at each 12 h of culture. At the first 12 h culture, *AIRE*^*−/−*^ spheroids had a cell count higher than *AIRE*^*WT*^ spheroids. Differently, the development of the *AIRE*^*WT*^ spheroids drew a classical growth curve, permitting the identification of the exponential, stationary, and decay phases. The slope of the *AIRE*^*−/−*^ spheroid growth curve indicates that in the absence of *AIRE*, the growth is increased at the beginning, and then the growth abruptly decreases. This suggests that in addition to controlling adhesion between cells, *AIRE* might influence the cell cycle of mTECs (Fig. 1B).

Besides the growth curve, we evaluated the cell viability during spheroid growth. The *AIRE*^*−/−*^ spheroids increased viability during the first 12 h and decreased thereafter, whereas the *AIRE*^*WT*^ progressively reached viability starting from 12 h and maintaining thereafter (Fig. 1C).

### *AIRE*^*−/−*^ spheroids influence the cell cycle kinetics and the cell death

After 12 and 24 h of adhesion, *AIRE*^*WT*^ and *AIRE*^*−/−*^ spheroids were dissociated and analyzed for the distribution of cells at the different cell cycle phases.

**Fig. 2:**
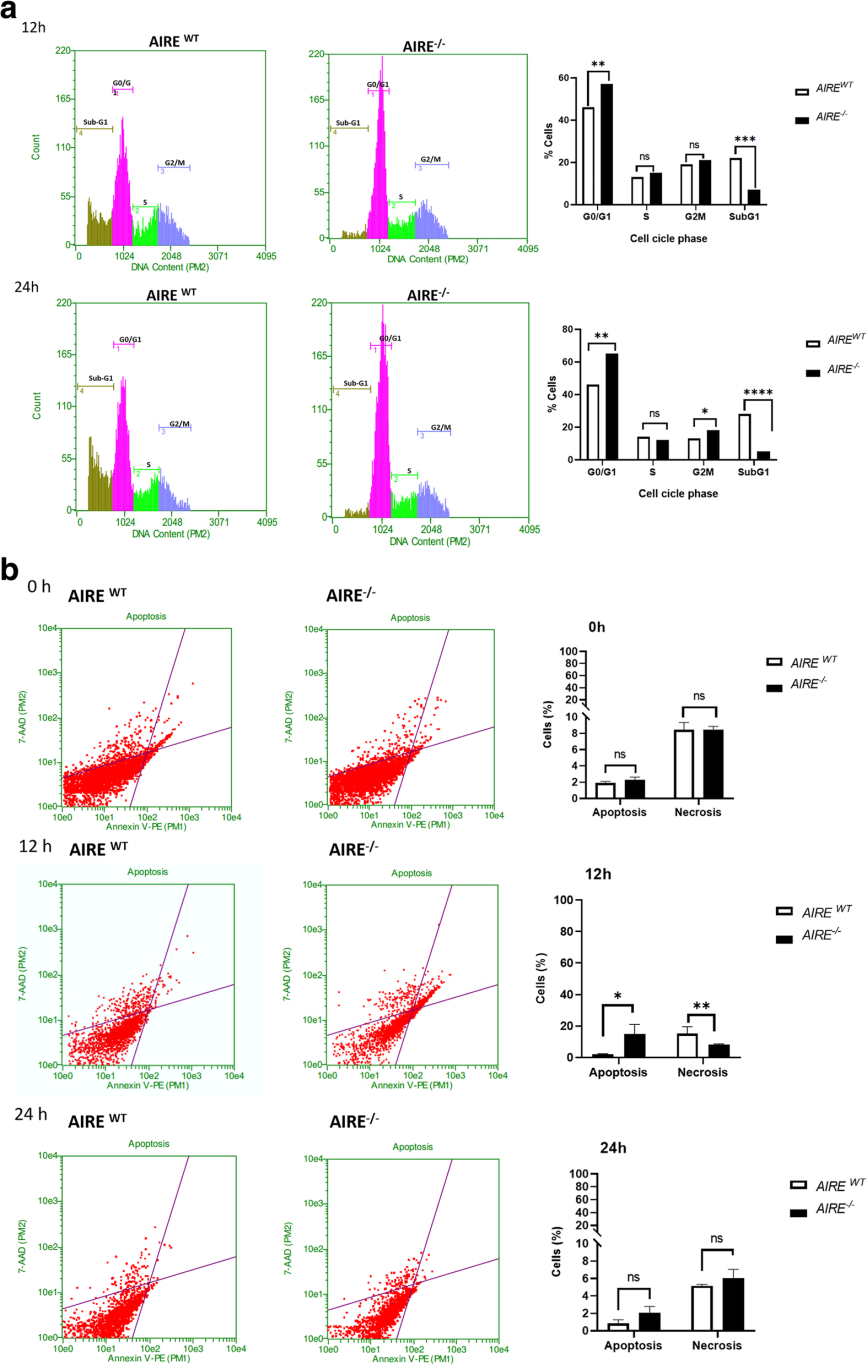
Cell cycle kinetics and spheroid cell death assay. **A** Cell cycle analysis in *AIRE* ^*WT*^ and *AIRE*^*- / -*^ spheroids was performed by flow cytometry using PI staining; cell proportions were determined for each phase of the cell cycle following 12 and 24 h of spheroid formation. Distribution of cells at different cell cycle and Sub-G1 phases following 12 and 24 h of spheroid formation (*N =* 3). Values are expressed as mean ± SEM from data obtained in three independent experiments. Statistical analysis: unpaired t test. ns = no significant difference; 12 h: GO/G1 = **p = 0,0011; S = ns; G2M = ns; SubG1 = ***p = 0,0002. 24 h: G0/G1 = **p = 0,0026; S = ns; G2M = *p = 0,0105; SubG1 = ****p = < 0,0001 respectively indicate statistically significant differences when compared to *AIRE* ^*WT*^ spheroids. **B** The cell death analysis was measured using the eBioscience™ Annexin V Apoptosis Detection Kit (Invitrogen™), which determines the number of viable cells and detects cell death by apoptosis or necrosis. Living cells are shown in the lower left part of the respective plots; dying/dead cells shift to the right and upper part of the plot (representative plots from one experiment). The graphics show the total numbers (%) of apoptotic and necrotic cells (mean values ± SEM from three experiments). Statistical analysis: unpaired t test. *p = 0,0240,**p = 0,0023; ns = no significant difference; respectively, indicate statistically significant differences for the comparison between *AIRE*^*- / -*^ and *AIRE*^*WT*^ spheroids

The cell counting performed at 12 h of spheroid formation indicated a significant increase in the percentage of cells at the G0/G1 phase, 46% in *AIRE*^*WT*^ and 57% in *AIRE*^*−/ -*^ cells, showing an accumulation of *AIRE*^−/−^ cells at G0/G1 (Fig. 2A). Interestingly, a significant reduction was observed in the sub-G1 fraction (7%) for *AIRE*^−/−^ cells compared to *AIRE*^*WT*^ (22%). Within 24 h of adhesion, a similar pattern was seen, with a significant increase in the percentage of cells at G0/G1 phase (65%) observed for *AIRE* ^−/−^ cells, as well as a lower percentage (5%) of the subG1 fraction compared to *AIRE*^*WT*^ (46% G0/G1; 28% SubG1). In addition, a significant increase (18%) in *AIRE*^−/−^ cells was observed at the G2/M transition compared to *AIRE*^WT^ (13%). Taken together, these results show that the absence of *AIRE* causes a significant alteration in the cell cycle progression, as demonstrated by the high number of cells undergoing the G0/G1 and G2 phases.

Since *AIRE* ^−/−^ spheroids showed lower percentage of sub-G1 fraction than *AIRE*^*WT*^, and since these fractions indicate a certain percentage of cell death, we analyzed the percentage of apoptosis and necrosis. After 12 h of spheroid formation, *AIRE*^*−/ -*^ cells showed a significant increase in the population of apoptotic cells (15%) compared to the *AIRE*^*WT*^(1.96%) (Fig. 2B). On the other hand, a significant reduction of necrotic cells was observed in *AIRE*^*−/−*^ (8.3%) spheroids analyzed after 12 h compared to *AIRE*^*WT*^(15.26%). At 0 h (prior to spheroid formation) and 24 h, we did not find significant differences between *AIRE*^*−/ -*^ and *AIRE*^*WT*^ spheroids regarding the percentage and the profile of cell death.

### Spheroid dead-cell center and spheroid morphology are impaired in the absence of *AIRE*

**Fig. 3:**
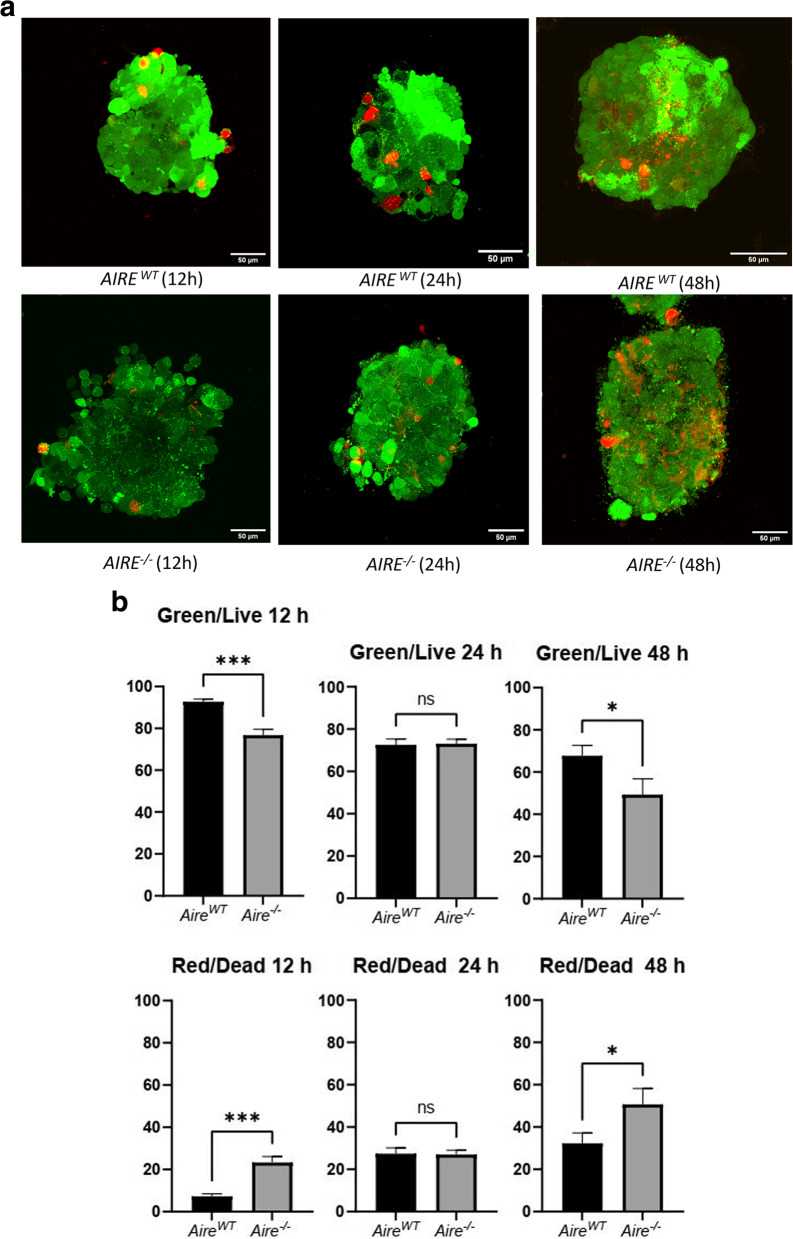
Spheroid confocal fluorescence microscopy. **A** Spheroids were stained with Live/Dead® Viability/Cytotoxicity kit reagents to detect live (green) or dead (red) cells of *Aire*^*WT*^ or *Aire*^*−/−*^ spheroids **B** Quantitative analysis of the fluorescence signal for live or dead cells comparing *Aire*^*WT*^ and *Aire*^*−/−*^ spheroids. Time-point values correspond to mean and ± SD from 20 spheroids analyzed. The MFI of the channels green and red was measured and compared through paired student t-test (*p*-value = *** 0,0008; ns 0,8097- 0,8196; * 0,0237 respectively). ns: no significant difference

In the center of the spheroids, dead cells were observed for both *AIRE*^*WT*^ or *AIRE*^*−/−*^ cells along the growth; however, *AIRE*^*−/−*^ spheroids exhibited an increased rate of dead cells along the growth, maximizing at 48 h, as shown in Fig. 3A. Quantitative analysis allowed us to determine the relative fluorescence intensity for both live (green-colored) and dead (red-colored) cells (Fig. 3B).

**Fig. 4:**
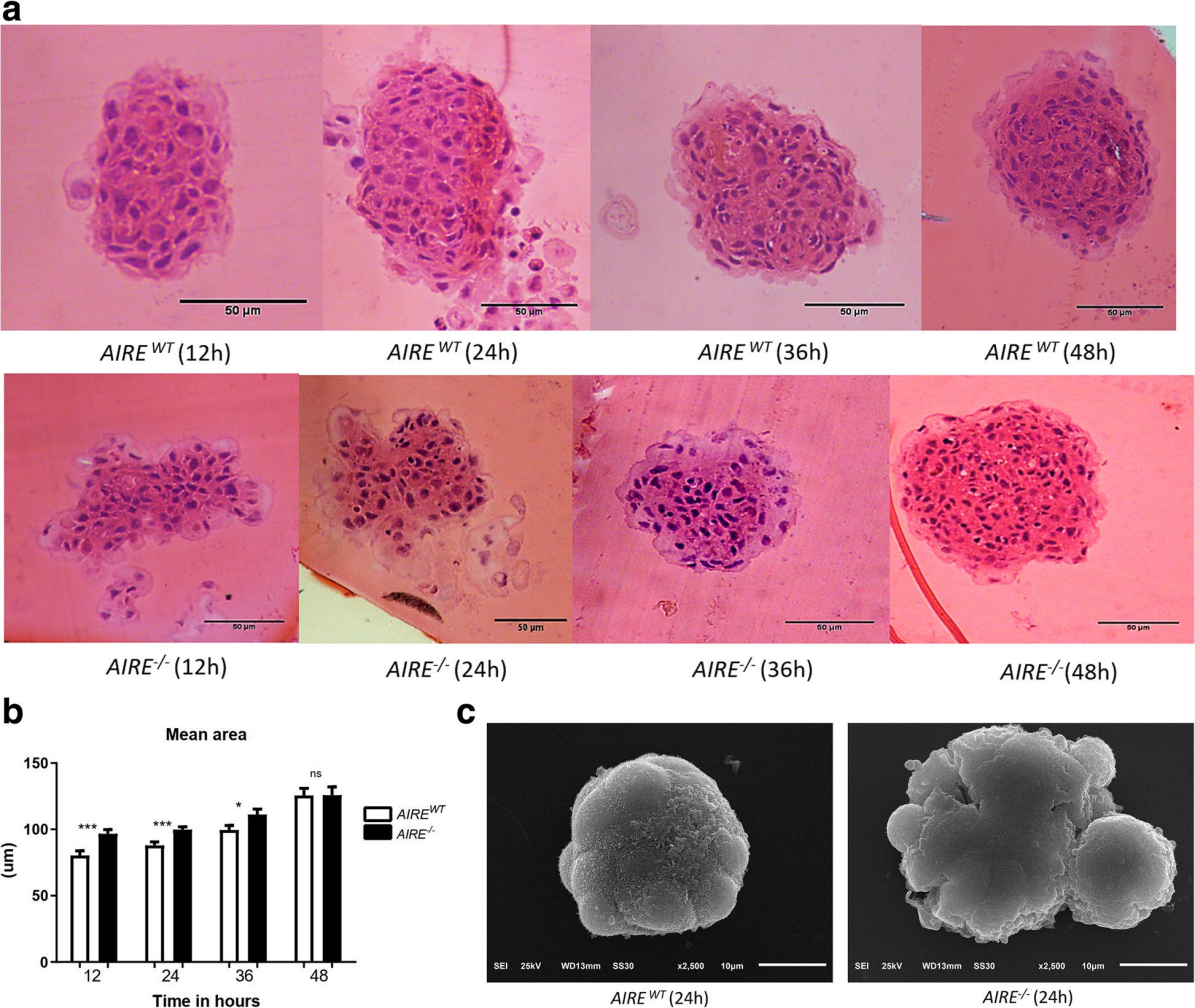
Spheroid morphology **A** Internal structure of the spheroids *Aire*^*WT*^ and *Aire*^*−/−*^ .Light microscopy (H&E staining) of histological analysis of spheroid growth from 12 to 48 h. In the upper part of the Figure is represented AIRE^WT^ spheroid at 12, 24,36 and 48 h of adhesion, showing regular round centers, exhibiting well-defined contours, whereas in the lower part of the Figure (AIRE^−/−^ spheroids), the cells present a lobule-like form, exhibiting ill-defined contours at 12 and 24 h of adhesion, resuming the regular round formation after 36 h of culture. **B** Area of spheroids during its growth from 12 to 48 h. The area was measured and compared through paired student t-test (p-value = *** 0.0007; *** 0.0008; * 0.0368; ns 0.9861 respectively). ns: no significant difference. **C** Scanning electron microscopy (SEM) of the spheroids *Aire*^*WT*^ and *Aire*^*−/−*^. A representative SEM images comparing 24 h *Aire*^*WT*^ vs. *Aire*^*−/−*^ spheroids. Representative images from 20 spheroids analyzed for each time-point. Scanning electron microscope (Jeol JSM -6610 LV), 5 kV acceleration voltage

The high-resolution optical microscopy of the 1 μm histologic cuts allowed the observation of the internal structure of spheroids. The *AIRE*^*WT*^ or *AIRE*^*−/−*^ spheroids showed significant differences in their areas, especially at the first 24 h of growth. Although *AIRE*^*−/−*^ spheroids present a larger area than *AIRE*^*WT*^ spheroids, *AIRE*^*−/−*^ cells are dispersed and compact similarly to *AIRE*^*WT*^ spheroids only after 24 h of adhesion (Fig. 4A, B).

Scanning electron microscopy (SEM) accessed the detailed external surface of the spheroids. Twenty-four-hour *AIRE*^*WT*^ spheroids were more compact, exhibiting a well-defined contour, while *AIRE*^*−/−*^ spheroids showed an irregular surface (Fig. 4C).

### The loss of *AIRE* impairs the strength of the cell-cell adhesion

**Fig. 5:**
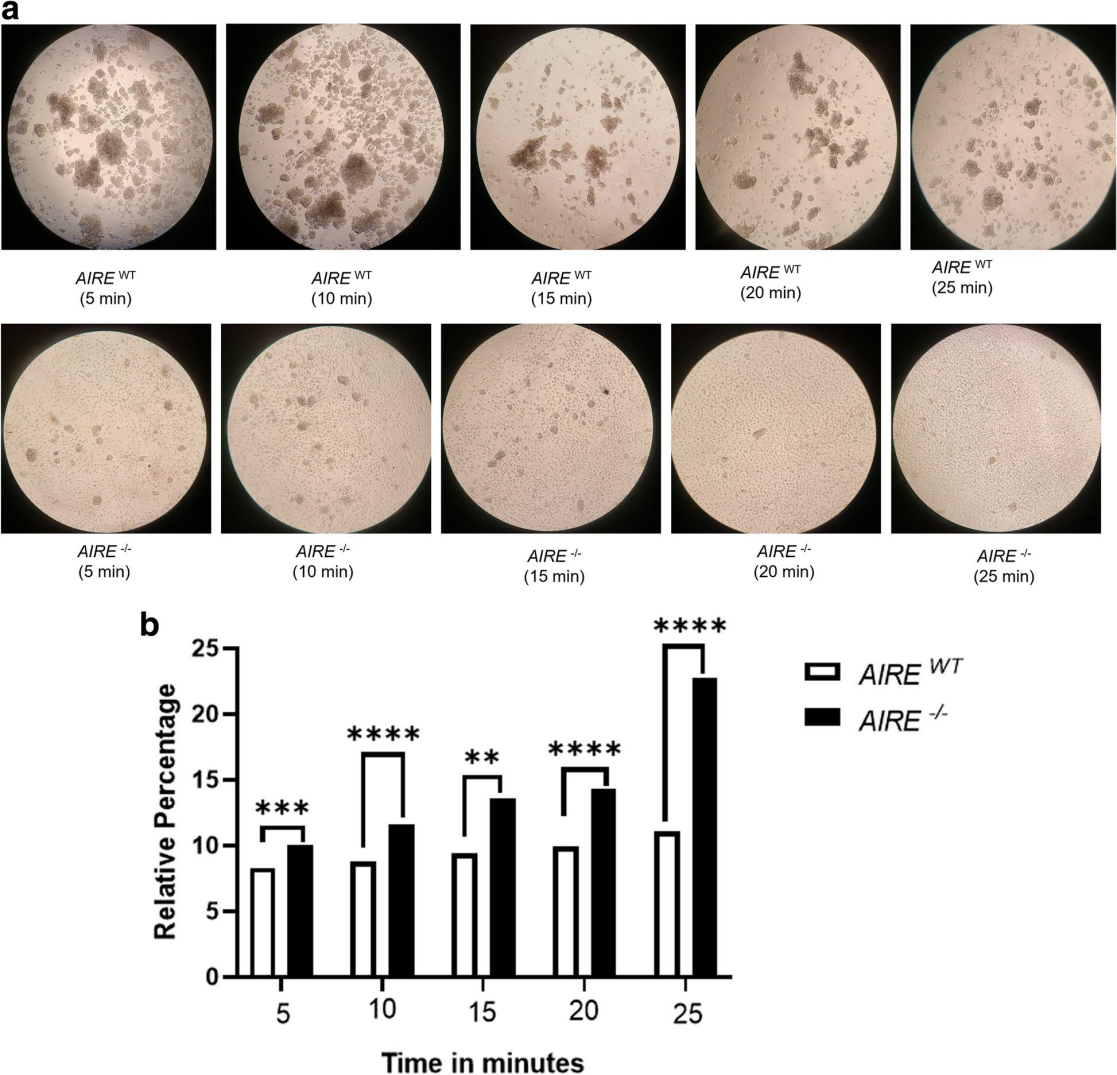
Dispase assay **A** Spheroids dissociation with dispase to evaluate the absence of AIRE in intercellular adhesion by the strength of dissociation **B** Quantification was obtained by counting the number of fragments of cell sheets as described in the Material and Methods section. At least three independent experiments were performed

To observe the strength of cell-cell adhesion, we induced an enzymatic spheroid disaggregation at 12 h of culture. Compared to *AIRE*^*WT*^, *AIRE*^*−/−*^ spheroids dissociated faster (Fig. 5A) and exhibited an increased number of disaggregated cells along the 25 min of culture (Fig. 5B), indicating that the lack of *AIRE* gene decreased the strength and spheroid adhesion.

### Phenotypic characterization of spheroid medullary cell surface markers

**Fig. 6:**
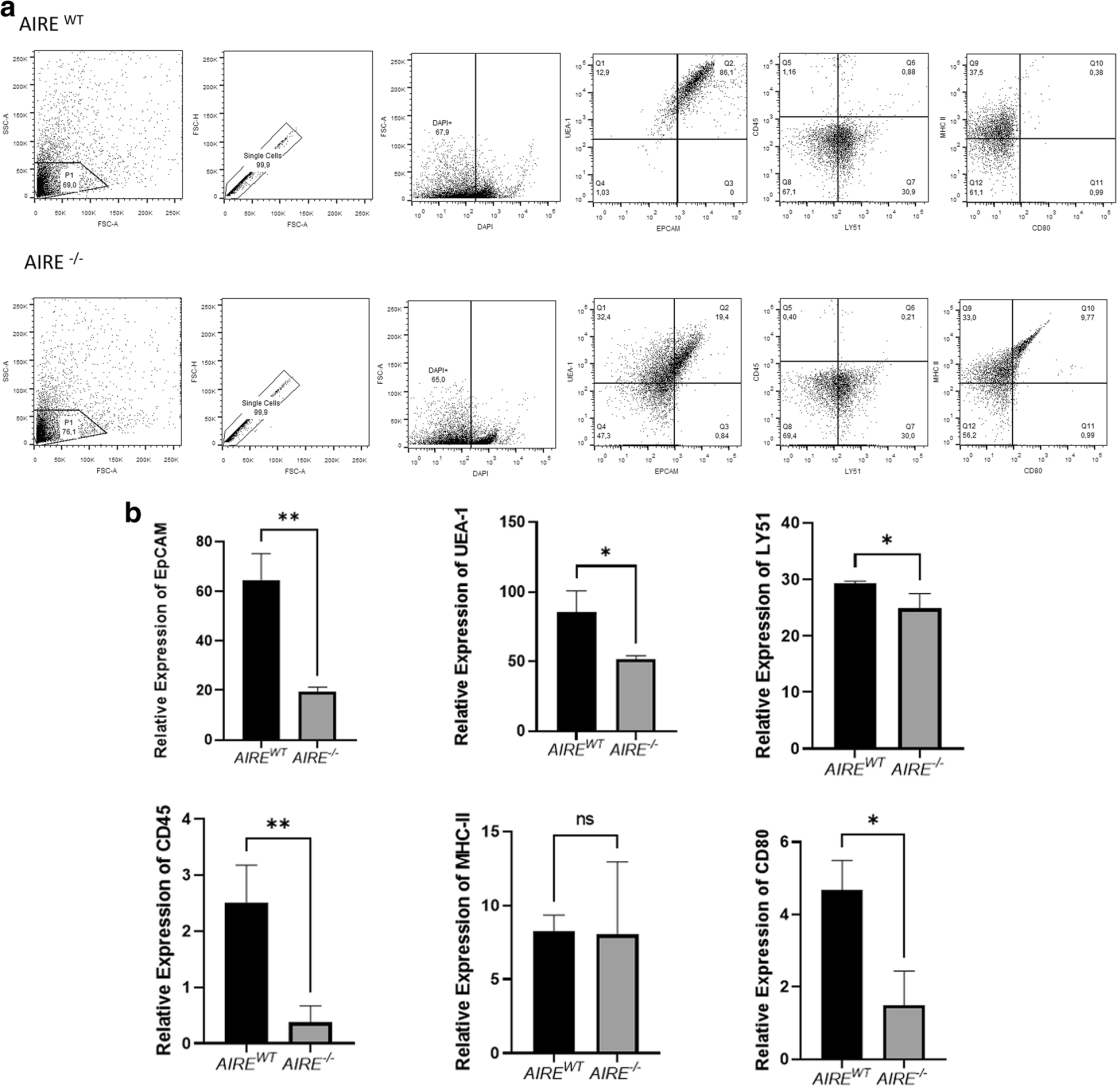
Phenotyping of spheroids **A** Flow cytometry analysis of phenotype of spheroids *Aire*^*WT*^ or *Aire*^*−/−*^ at 12 h of adhesion by the expression of a viability marker and cell surface markers: UEA-1; Epcam; CD45; Ly51; MHCII and CD80 (representative figure from nine independent determinations). **B** Quantification of the markers by Prism GraphPad. Data shown (mean ± SD) are from nine independent determinations. The significant difference between *AIRE* ^*WT*^ and *AIRE*^*−/−*^ was analyzed by the unpaired t-test Epcam: **p = 0.0019; LY51: *p = 0.0471; UEA-1: *p = 0.0171; CD45: **p = 0.0069; MHCII: ns; CD80: *p = 0.0114. no significant difference

*AIRE* ^*WT*^ spheroids showed: i) the characteristic CD45^−^ Ly51^*−*^ medullary phenotype, observed in 67.1% of mTECs, ii) 86.1% of the cell population expressed EpCAM and UEA-1, iii) 0.38% of cells were double positive for CD80^+^ MHC-II (Fig. 6A). In contrast, the *AIRE*^*−/−*^ spheroids featured: i) the characteristic CD45^−^ Ly51^−^ medullary phenotype in 69.4% of mTECs, ii) 19.4% of the cell population expressed EpCAM^+^ and UEA-1^+^, iii) 9.77% of cells were double positive for CD80^+^ MHC-II^+^ (Fig. 6A). Comparing the expression of each marker separately, we observed that the *AIRE*^*WT-,*^ *AIRE*^*−/−*^ spheroids exhibited: i) decreased expression of EpCAM, UEA-1, CD45, CD80 molecules ii) a closely similar expression of MHCII and Ly51 molecules (Fig. 6B).

### Spheroid RNA-Seq analysis

**Fig. 7:**
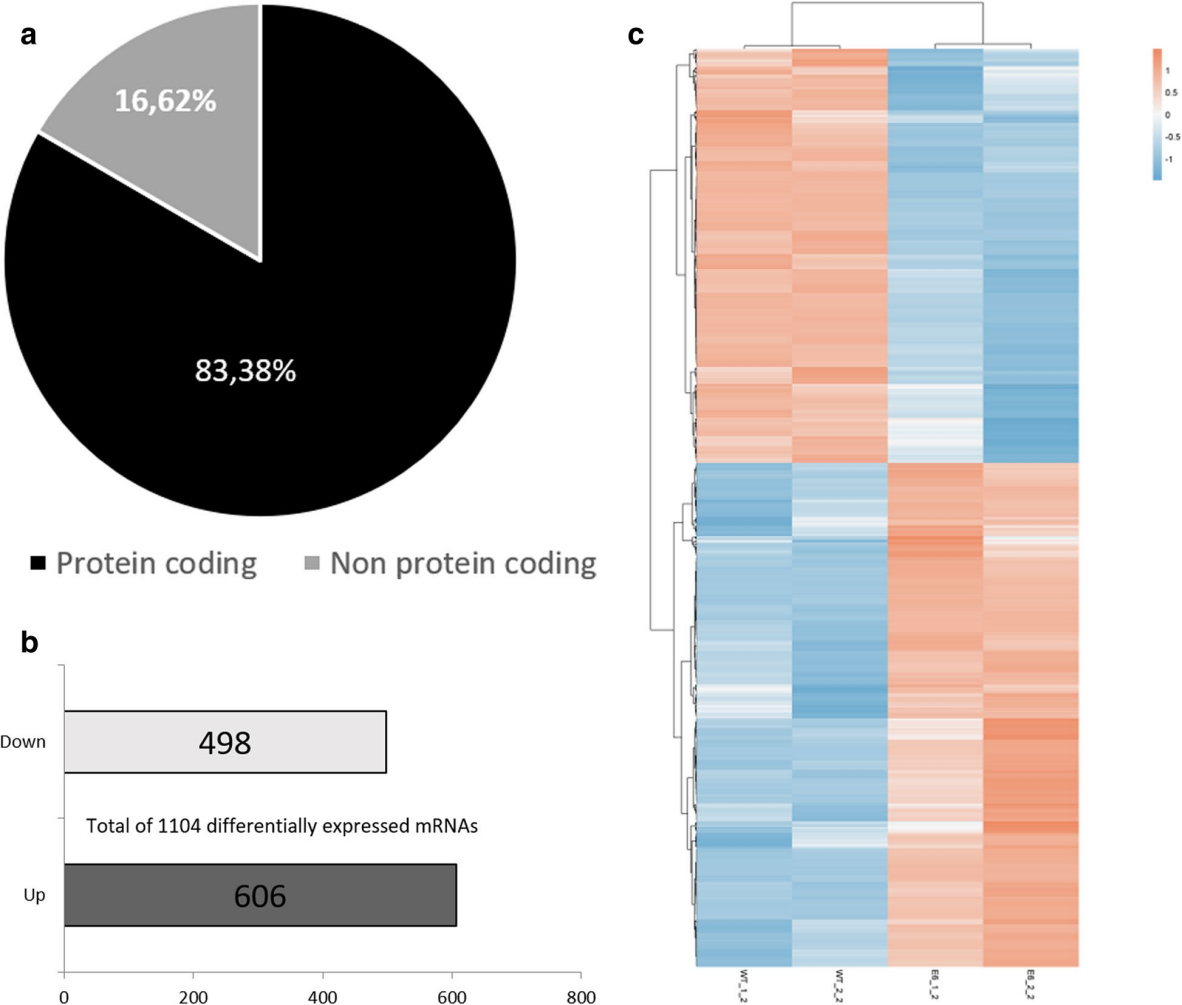
Transcriptome (mRNAs) expression profiles of spheroids. (**A**) Quantitative analysis of differentially expressed genes grouped to its coding or noncoding function (fold change ≥. 2.0, p-value ≤0.05). (**B**) Number of differentially expressed protein coding genes (fold change ≥. 2.0, p-value ≤0.05). (**C**) Transcriptome (mRNAs) expression profiles of spheroids AIRE^WT^ compared to AIRE^−/−^. The spheroid cells’ total RNA samples were analyzed through RNA-seq, which allowed identify the differentially expressed mRNAs. The dendrograms and unsupervised heat-maps were obtained using the R platform, the cluster and tree view algorithm considering 2.0-fold-change and 0.05 false discovery rate. Heat-map legend: red = upregulated, blue = downregulated (Pearson’s correlation metrics)

Comparative transcriptome analyses were performed for *AIRE*^*WT*^ and *AIRE*^*−/−*^ spheroids at 12 h. We identified a set of 1210 differentially expressed (DE) mRNAs, of which approximately 83% (1104) corresponded to protein-coding mRNAs, and about 17% (106) corresponded to non-protein-coding RNAs (Fig. 7A). Among the 1104 DE mRNAs, 606 were upregulated and 498 downregulated (Fig. 7B). Figure 7C shows the hierarchical clustering of the DE mRNAs.

**Fig. 8:**
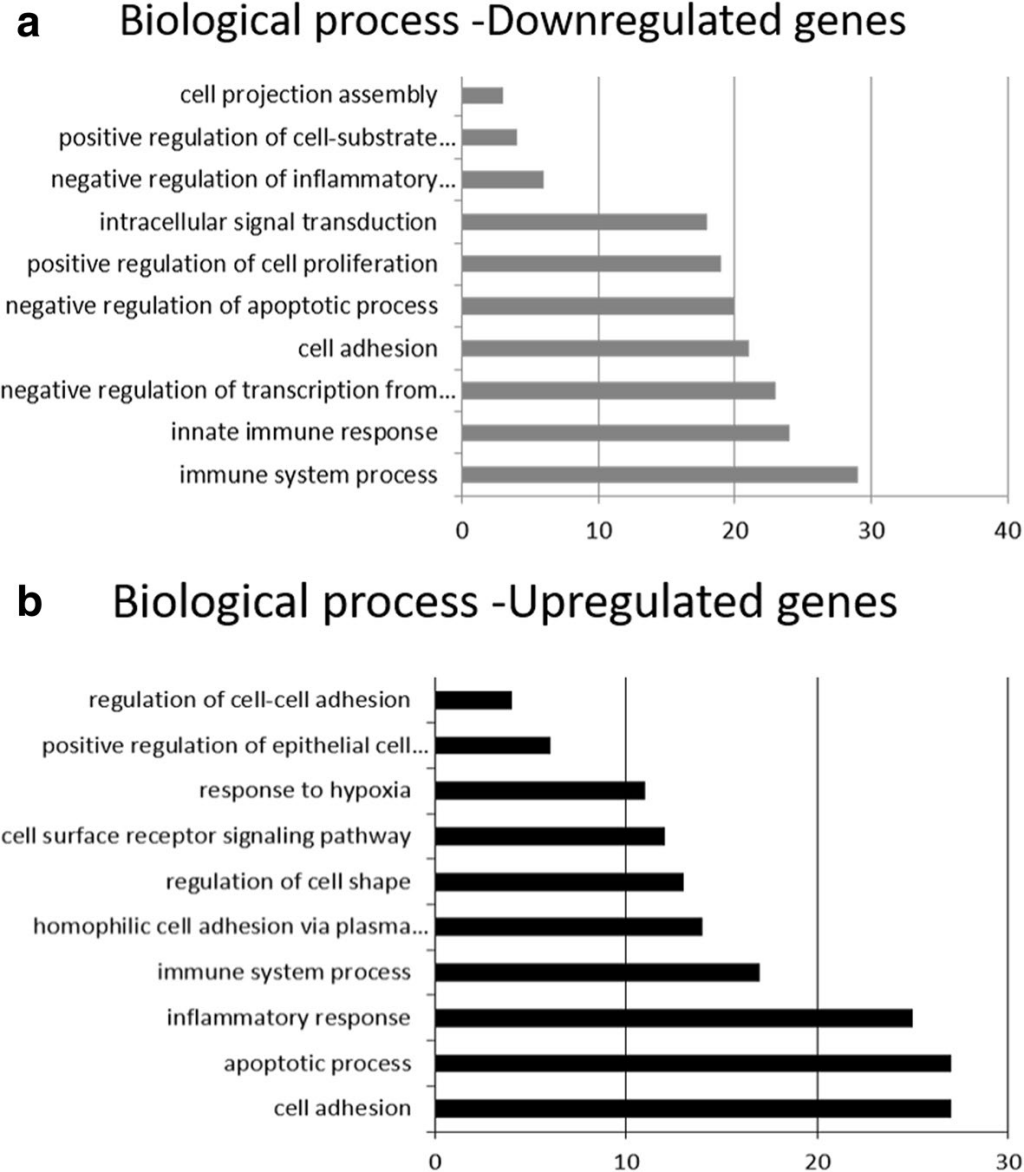
The top 10 biological process involved in spheroid profile comparing *AIRE*^*WT*^ vs. *AIRE*^*−/−*^. Functional annotation for modulated down **A** or upregulated **B** mRNAs in spheroids. The functional categories were identified by using DAVID genome database platform according to GO biological processes (BP). The rank is based on the enrichment score, which represents mean *p*-value. Only those mRNA-groups yielding < 0.05 Benjamini corrected *p*-value and contained at least five mRNAs are considered to be significant (DAVID Bioinformatics Resources Platform 6.8 Database, score < 0.05)

**Fig. 9:**
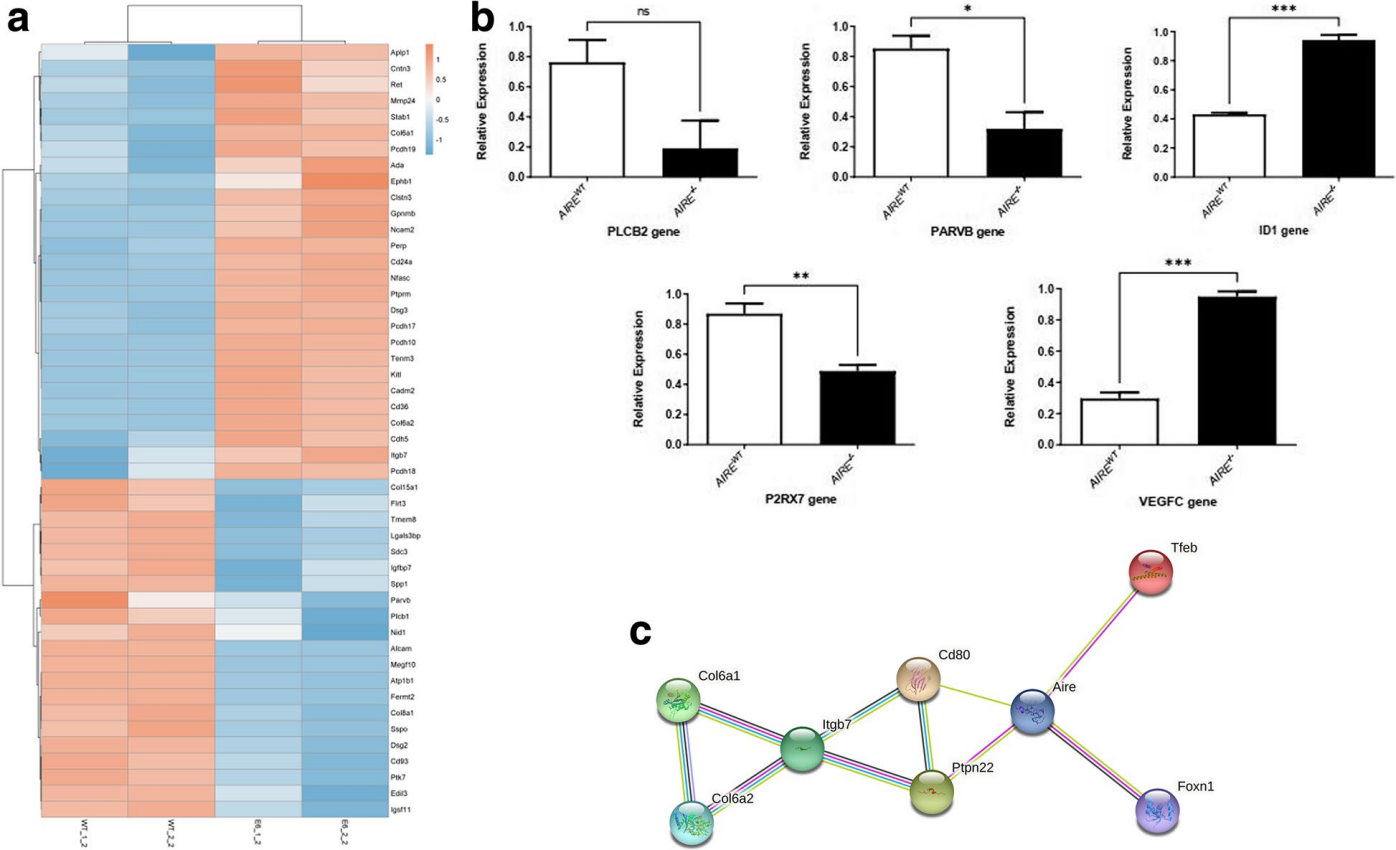
Analysis of RNA sequencing data. **A**- Heat-map showing the large-scale expression profiling of differentially expressed mRNAs involved in cell adhesion pathway. Unsupervised heat-maps and dendrograms were constructed using the R platform. Heat-map legend: red = upregulated, blue = downregulated (Pearson’s correlation metrics, fold change ≥2.0 and false discovery rate (FDR) < 0.05). **B** Evaluation of the relative expression level of the mRNAs PLCB2, PARVB, P2RX7, VEGFC and ID1 by RT-qPCR. Difference between groups was analyzed by Unpaired-t test, comparing data from *AIRE*^*WT*^ vs. *AIRE*^*−/−*^ − *p* value = PLCB2 = ns 0,0738, PARVB = *0,0184, ID1 = ***0,0001, P2RX7 = ** 0,0089, VEGFC = *** 0,0002. **C** Genetic Interaction network. A network of 7 proteins identified with interaction with Aire. All nodes represent first order interaction. Colored edges convey status of predicted network edge correspondingly cyan, curated database; magenta, experimentally determined; forest green, gene neighborhood; red, gene fusion; navy blue, gene co-occurrence; lawn green, text mining; black, co-expression; lavender indigo, protein homology. Node color signifies protein functionality. Additional nodes are considered based on prediction score ≥ 0.9 (for more details, refer to STRING database)

Figure 8 shows the differences observed for the top-ten biological functions of downregulated (Fig. 8A) and upregulated mRNAs (Fig. 8B). Figure 9A shows the differentially expressed genes involved in the positive regulation of epithelial cell proliferation. Figure 9B confirms by RT-PCR the differential expression of some top-ten DE mRNAs, including the downregulated *PARVB* that encodes a protein associated with the cell adhesion pathway and the *PLCB2* and *P2RX7* that are involved in the calcium signaling pathway in *AIRE*^*−/−*^ when compared to *AIRE*^*WT*^ spheroids. In contrast, the *VEGFC* and *ID1* transcripts, associated with the regulation of epithelial cell proliferation pathway, were upregulated in *AIRE*^*−/−*^ spheroids. We took advantage of the use of the STRING algorithm, which establishes interactions among the encoded proteins based on the validated data retrieved from the literature, to understand the interaction of these genes. Figure 9C shows that the Aire protein may interact with other proteins associated with the immunological response (Foxn1 and CD80), autoimmune disorders (Ptpn22), adhesion receptor (Itgb7), collagen (Col6a1 and Col6a2), and apoptosis (Tfeb).

## Discussion

Considering that: i) the *AIRE* gene, besides controlling the expression of genes that encode tissue-specific antigens (TSAs) [7, 9, 14], also controls the expression of adhesion molecule genes [17, 18], ii) studies regarding the role of *AIRE* in the negative selection have primarily focused on the interaction mTEC-thymocyte, and iii) little attention has been devoted to the role of *AIRE* on the mTEC-mTEC adhesion, in the present study we used 3D spheroids to evaluate the role of the *AIRE* gene on the mTEC-mTEC adhesion. We showed that the absence of the *AIRE* gene: i) disorganizes the 3D structure of mTEC spheroids, ii) increases the proportion of cells at the G0/G1 phase of the cell cycle, iii) increases the rate of mTEC apoptosis, iv) decreases the strength of mTEC-mTEC adhesion, v) promotes a differential regulation of mTEC classical surface markers, and vi) modulates genes encoding adhesion and other molecules.

Several strategies have been developed to study the functional thymic properties, including ex vivo reaggregation thymic organ culture (RTOC) [19, 20], 2D mTEC-thymocyte adhesion [17, 18], transwell thymocyte migration [27], 3D fetal thymus organ culture (FTOC) [28], and 3D organotypic culture [23]. Considering that the 3D intrathymic environment is necessary for the development of a functional thymus scaffolding, development of thymic epithelial cells, chemoattraction of pro-T-cells, commitment to the T lineage, and generation of a repertoire of T-cell receptors (TCR) and natural T regulatory cells [29–31], an adequate thymus structure is mandatory to establish organ functionality.

Since the consequences of *AIRE* deletion in the intercellular adhesion remain to be elucidated, we studied the interaction between mTECs when these cells were seeded in a non-adherent substrate such as agarose, where 3D spheroids may be observed at the various stages of development. Once in an agarose substrate, mTECs aggregate and adhere to each other to form spheroids, which may be helpful for the study of mTEC-mTEC adhesion. Then, we evaluated the consequences arising from the absence of *AIRE*, evaluating the: i) mTEC-mTEC physical interaction at regular periods, ii) growth curve of mTEC spheroids, iii) dynamics of 3D mTEC spheroid formation and spheroid dissociation, iv) cell-cycle phases and the rate of apoptosis/necrosis, v) morphology of mTEC spheroids, vi) expression of surface mTEC molecules and vii) gene expression profiles, comparing *AIRE* ^WT^ and *AIRE*^*−/−*^ cells.

We reported that *AIRE* interferes with the early process of spheroid formation, since its absence yielded a delay on the spheroid growth and on cell viability (Fig. 1A-C). Although *AIRE* is a pro-apoptotic factor in mature mTECs [32], in this study we observed that during the 3D spheroid formation *AIRE*^−/−^ spheroids presented increased apoptosis rate. Indeed, the absence of *AIRE* produced a significant impact on the progression of cells through the cell cycle (Fig. 2A), mainly at 12 h. The higher percentage of cells undergoing the G0/G1 phase in *AIRE*^−/−^ spheroids are possibly related to the higher percentage of apoptotic cells when compared with *AIRE*^WT^ spheroids (Fig. 2B). These results suggest that the lack of *AIRE* function may affect the cascade of signaling pathways, leading to alterations in the cell cycle progression (checkpoint mechanism) and apoptosis induction in proliferating cells. Corroborating this idea, we observed that the several genes related to apoptotic processes were upregulated in the *AIRE*^−/−^ spheroids, including the *PERP, PDCD4, TNFRSF21, SLC5A8, S100A9, PPID, CYCS, CARD14, ALDOC, CKAP2, STK17B, POLB, CDCA7, GADD45B, APLP1, CASP1, CASP4, BNIPL, TNS4, AIM2, IL24, STEAP3, FAM3B, SRGN, EPB41L3, EPHA7, FGFR2* and *S100A8* genes. Notably, the *CDCA7* and *GADD45B* genes are involved in cell division and mediate Fas-induced apoptosis, respectively.

Regarding the physical structure of the 3D spheroids, dead cells were observed in the center areas of both spheroid types; however, the number of these cells progressively increased along time, predominating at 48 h in *AIRE*^*−/−*^ spheroids (Fig. 3A-B). Besides, in the model system of this study, it was possible to observe a distinct morphology of the mTEC interactions; i. e., the *AIRE*^*WT*^ forms a typical spheroid at 12 h, whereas the *AIRE*^*−/−*^ initially forms an ellipsoid form instead of a spheroid shape (Fig. 4A/B). Noteworthy, the scanning electron microscopy showed that *AIRE*^*WT*^ spheroids are well-compacted with a well-defined contour. In contrast, *AIRE*^*−/−*^ spheroids exhibited an irregular shape and ill-defined surfaces (Fig. 4C), indicating that *AIRE* influences the internal and external spheroid structure.

Since the 3D *AIRE*^*−/−*^ spheroids exhibited a delayed formation at 12 h, as shown in Fig. 1A, we also evaluate the strength of spheroid dissociation, using a dispase assay. Indeed, *AIRE*^*−/−*^ spheroids dissociated faster than *AIRE*^*WT*^ spheroids and exhibited increased number of disaggregated mTECs (Fig. 5 A-B). Taken in concert, these in vitro results corroborate the in vivo finding of a disorganized thymic medulla in *AIRE*^*−/−*^ mouse [22] and add information regarding the dynamics of mTEC growth and mTEC-mTEC interactions.

In addition to the spheroid structure, we also evaluate the role of *AIRE* on the mTEC surface markers (Fig. 6A/B). Both *AIRE*^*WT*^ or *AIRE*^*−/−*^ spheroids maintain their characteristic CD45^−^ Ly51^−^ medullary profile, i.e., confirming that they are not hematopoietic cells (CD45^+^) nor cTECs (Ly51^+^). The absence of *AIRE* decreased the expression of the EpCAM and UEA-1 cells, which are classical markers for epithelial cells and medullary thymic cells, respectively. Noteworthy, the expression of double positive (CD80^+^ MHCII^+^) was increased in *AIRE*^*−/−*^ spheroids (0.38% in *AIRE*^*WT*^ to 9.77% in *AIRE*^*−/−*^). Three major stages can be observed along mTEC maturation: i) the immature mTEC presents MHC-II^low^, CD80^low^ and Aire^low^, ii) during TRAs, mTECs exhibit their mature stage, presenting MHC-II^high^, CD80^high^ and Aire^high^, and iii) at the post-Aire stage, mTECs are MHC^intermediary^, CD80^intermediary^, accompanied by an increased rate of apoptotic cells [2]. As observed in Fig. 6A/B, the *AIRE*^*WT*^ spheroids present features of an immature mTEC constitutively exhibiting low expression of *AIRE*, as previously reported by our group [17]. In contrast, *AIRE*^*−/−*^ spheroids present an intermediate expression of CD80 together with increased rate of dead cells, features that are characteristic of the post-Aire stage. These findings indicate that in the absence of Aire, mTECs present a terminally differentiated mTEC during the first 12 h of 3D spheroid culture, which may affect the interaction with thymocytes.

Considering that *AIRE* controls more than 3300 genes in mTECs [33], we initially evaluated the mRNA transcript profiles of *AIRE*^*WT*^ or *AIRE*^*−/−*^ spheroids at 12 h of mTEC-mTEC adhesion, showing a distinct pattern of gene expression (Fig. 7A-C). Among the DE mRNAs, we identified genes associated with cell adhesion, positive regulation of cell proliferation, apoptotic process, and response to hypoxia (Fig. 8A/B). Among the DE mRNAs related to cell adhesion, we observed a set of transcripts that encode proteins belonging to the cadherins (*PCDHGB7*), collagen (*COL15A1*), integrin (*CIB3*), fibronectin (*FLRT3*), or extracellular matrix protein families (*MMP19*), which were downregulated in *AIRE*^*−/−*^ spheroids and are putatively involved on the dysregulation of the spheroid formation, as observed in this study. In contrast, *VEGFC* and *ID1* genes, involved in the positive regulation of epithelial cell proliferation, were upregulated (Fig. 9A/B), a finding that may be associated with the increased number of cells at 12 h of growth (Fig. 1B). The network generated by the STRING tool (Fig. 9C) revealed that Aire interacts with important proteins involved in the immunological response, autoimmune disorders, adhesion receptor, formation of collagen, and apoptosis, indicating that the lack of Aire impairs the 3D spheroid conformation.

Noteworthy, we observed a set of repressed mRNAs associated with the calcium signaling pathway, and among these, the *ADCY3, ADCY1, NOS1, P2RX7, ADRB2, PLCB4, ATP2B4, ATP2A3, P2RX3, CACNA1G, ADRA1B, PLCB1, PLCB2*, and *F2R* transcripts are associated with the intercellular calcium waves. Since intercellular communication used by mTECs is mediated by intercellular calcium waves, requiring functional gap junctions and P2 receptors [34, 35], these genes are potential candidates to be further studied.

Even though operating through different ways, *AIRE* and mTEC-mTEC adhesion culminate into related activities that regulate a cascade of mRNAs that encodes cell adhesion molecules and other relevant molecules that maintain the 3D spheroid formation, opening new perspectives for studies of molecular mechanisms that control the 3D thymic medulla organization and mTEC adhesion and communication.

## Conclusion

While most studies have focused on the mTEC-thymocyte interaction to evaluate the negative selection, this study focused on the role of *AIRE* on the mTEC-mTEC interaction as the primary step to support the adequate thymic medullary structure. Considering the 3D spheroid model, this study reported that the absence of *AIRE* disorganizes the 3D structure of mTEC spheroids, promotes a differentially regulation of mTEC classical surface markers, and modulates genes encoding adhesion and other molecules.

## Materials and methods

### Medullary thymic epithelial cell lines

We use the *AIRE*^*WT*^ murine (*Mus musculus*) mTEC 3.10 line (EpCAM^+^, CD45^−^, Ly51^−^, UEA-1^+^), as previously described [17, 36, 37], and the *AIRE*^*−/−*^ mTEC 3.10E6 cell clone. The mTEC 3.10E6 clone obtained by the CRISPR-Cas9 system [18] was characterized as a carrier of indel mutations affecting both *AIRE* alleles (compound heterozygous). In the *AIRE* allele 1, there were two types of mutations: a T > G substitution (mRNA nucleotide, nt, position 351), followed by a nine-bp deletion (GCTGGTCCC, mRNA nt positions 352–360) that transcribed a 1647 nt *AIRE* mRNA. In allele 2, a single G deletion at mRNA nt position 352 transcribed a 1655 nt *AIRE* mRNA. As both alleles of the mTEC 3.10E6 clone produced a nonfunctional *AIRE* protein, they were considered knockout; i. e., *AIRE*^*−/−*^*.*

### Spheroid formation

We use a precast agarose mold with non-adherent 600 μm diameter microwells, making the mTEC cells adhere once seeded in these compartments. The *AIRE*^*WT*^ mTEC 3.10 and the *AIRE*^*−/−*^ mTEC 3.10E6 cell lines were initially cultured as monolayers in RPMI 1640 medium (Gibco, Darmstadt, Germany) supplemented with 10% inactivated fetal bovine serum in 75 cm^2^ polystyrene plastic bottles (Corning, New York, NY) in an incubator at 37 °C with 5% CO_2_ atmosphere. After acquiring confluence, mTECS were trypsinized and seeded in agarose molds, using 2% low electroendosmosis agarose (Sigma-Aldrich, Saint Louis, MO), sterilized in 70% ethanol, washed twice in sterile PBS, and irradiated under germicidal UV light for 15 min. Spheroid growth was observed through a Cytosmart® (Lonza Group AG, Basel, Switzerland) inverted microscope to produce a real-time movie of the culture.

### Growth curve

To better characterize the 3D culture model, we quantified the cells that form the spheroids at different time points (determinations at every 12 h). At each time point, the spheroids were removed from the agarose microwells and dissociated using trypsin. Isolated cells were counted using a Cellometer® Auto T4 Bright Field Cell Counter (Nexcelom Bioscience, Lawrence, MA). Triplicates were performed for each time point. We have thus drawn a spheroid growth curve to identify the exponential, stationary growth, and decline phases.

### Analysis of cell cycle

Analysis of cell cycle kinetics was performed by flow cytometry using the Guava cell cycle analysis. *AIRE*^*WT*^ and *AIRE* ^*−/−*^ spheroids were stained with propidium iodide (Sigma-Aldrich), a fluorescent nuclear marker. After 12 and 24 h of spheroid formation, the samples were collected, dissociated with ACCUMAX (Sigma-Aldrich) and stored at − 20 °C in 70% cold ethanol kept for 18 h. The samples were then resuspended in 200 uL of the propidium iodide (5 μg/mL) solution and maintained for 30 min at 37 °C. The cells were analyzed with a Guava EasyCyte Mini System flow cytometer (Merck Millipore, Burlington, MA) using the Guava CytoSoft software, version 4.2.1 (Merck Millipore). At least three independent experiments were analyzed.

### Cell death assay

*AIRE* ^*WT*^ and *AIRE* ^*−/−*^ cells (2 × 10^5^ cells/well) prior to spheroid formation (0 h) and spheroids with 12 and 24 h of adhesion were stained with annexin V-PE and 7AAD. After disassociation of the spheroids with ACCUMAX, cells were labeled with the eBioscience™ Annexin V Apoptosis Detection reagents (Invitrogen™, Waltham, MA), according to the manufacturer’s protocol. Analysis of apoptosis and necrosis was carried out on a Guava PCA Instrument using CytoSoft Software. At least three independent experiments were analyzed.

### Live-dead assay

We used the LIVE/DEAD® Viability/Cytotoxicity Kit (Thermo-Fisher, Waltham, MA) to assess the proportion of live and dead cells in the spheroids, following the manufacturer’s instructions.

### Histological analysis of spheroids

The spheroids were fixed in 10% formaldehyde buffered in PBS, then dehydrated, passing through a battery of aqueous ethanol solution (75 to 100% ethanol) and included in historesin (HistoResin standard kit, Biosystems Switzerland AG, Muttenz, Switzerland). The resin blocks were cut to 1 μm thickness and deposited on microscope slides. After deparaffinization and rehydration, the slides were stained with hematoxylin-eosin (H&E) for further microscopic examination of the spheroid morphology.

### Scanning electron microscopy (SEM)

Spheroids were fixed with 1.6% glutaraldehyde in 0.2 M buffered (pH 7.4) sodium cacodylate overnight at 4°C, then washed three times with 0.2 M sodium cacodylate buffer (pH 7.4). Spheroids were immersed into 1% osmium tetroxide (Sigma-Aldrich) for secondary fixation in 0.2 M buffered (pH 7.4) sodium cacodylate. Fixed spheroids were subsequently dehydrated in 25-100% ethanol and immersed in hexamethyldisilazane (HMDS, Sigma-Aldrich) for 10 min at room temperature. The spheroids were freeze-dried until the HMDS had evaporated, mounted, coated with palladium gold, and examined under a scanning electron microscope (Jeol JSM − 6610 LV, Hitachi High-Tech America, Tokyo, Japan) at an acceleration voltage of 5 kV. The SEM procedures were done at the Electron Microscopy Facility, Ribeirão Preto Medical School, University of São Paulo, Ribeirão Preto, SP, Brazil.

### Spheroid disaggregation assay

*AIRE* ^*W*T^ and *AIRE* ^*−/−*^ spheroids were cultured and after 12 h of adhesion the spheroids were incubated with dispase (Roche Diagnostics, Basel, Switzerland) for a maximum of 30 min to release cells as monolayers. Every 5 min released monolayers were carefully washed with PBS twice and subject to mechanical stress by pipetting with 1 mL pipettes, and the cells were counted using the Cellometer® Auto T4 Bright Field Cell Counter (Nexcelom Bioscience, Lawrence, MA). Triplicates were performed for each time point.

### Phenotyping by flow cytometry

We used a BD FACSymphony™ A1 Cell Analyzer (Becton Dickinson, Franklin Lakes, NJ) flow cytometer to evaluate the medullary phenotype of *AIRE*^*WT*^or *AIRE*^*−/−*^ spheroids. A sample of 1 × 10^6^ mTECs separated from spheroids were labeled with 1:250 dilution of anti-mouse CD45-PerCP, anti-mouse CD326 (EpCam), anti-mouse Ly51-PE (BD Biosciences, San Jose, CA), anti-mouse CD80-APC, and anti-mouse MHCII-PE antibodies in a final volume of 200 μL of cell suspension. Labeling was also performed with a 1:250 dilution of Lectin agglutinin I (*Ulex europaeus* UEA-I)-FITC (Vector Labs, Burlingame, CA) and the viability marker DAPI- BD Pharmingen™ .

### Total RNA preparation and cDNA synthesis

Total RNA of *AIRE*^*WT*^ or *AIRE*^−/−^ spheroids was prepared using the mirVana kit® (Ambion, Austin, TX) according to the manufacturer’s instructions. Microfluidic electrophoresis evaluated RNA integrity using Agilent RNA 6000 nano chips and an Agilent 2100 Bioanalyzer (Agilent Technologies Santa Clara, CA). Only RNA samples that were free of proteins and phenol and had an RNA Integrity Number (RIN) ≥7.0 were selected for cDNA synthesis or RNA sequencing. For cDNA synthesis, the SuperScript® reverse transcriptase enzyme (Invitrogen Corporation, Carlsbad, CA) was used according to the manufacturer’s instructions.

### Transcriptome analysis through RNA-Seq

We followed a protocol previously described by St-Pierre et al. [11]. Briefly, paired-end (2 × 150 bp) sequencing was performed by an Illumina HiSeq 2500 sequencer (Illumina, San Diego, CA), using a TruSeq Stranded Total RNA Library Prep Kit (Illumina). The quality of raw FASTQ sequences was first analyzed through a FASTQC program (https://www.bioinformatics.babraham.ac.uk/projects/fastqc/). Then, FASTQ sequences were mapped to the *Mus musculus* reference genome (mm21) using the STAR 2.5 Spliced Aligner program (https://github.com/alexdobin/STAR), which outputs BAM file containing the sequences and their genomic references and a GTF file with gene annotations used for further determinations of the number of reads per transcript through the HTSeq Count program (http://htseq.readthedocs.io). For each RNA sample analyzed, we recovered a list of genes and their respective number of transcripts that served as input for determinations of the differentially expressed (DE) mRNAs through the EdgeR package (https://bioconductor.org/packages/release/bioc/html/edgeR.html) within the R platform (https://www.r-project.org). EdgeR calculates the fold change (FC) for each mRNA, considering a contrast matrix for a given experimental condition. This study defined WT as a contrast variable and as DE the mRNAs exhibiting a *p*-value < 0.05 and a false discovery rate (FDR Benjamini–Hochberg correction) FC ≥ 2.0. The DE mRNAs were hierarchically clustered, and a heat map was constructed to evaluate the expression profiling. The raw RNA-seq data from this work is available on Gene Expression Omnibus (GEO) (https://www.ncbi.nlm.nih.gov/geo/) under accession number PRJNA763914.

### Functional enrichment of differentially expressed mRNAs

The list of the DE mRNAs was analyzed in terms of functional enrichment through the Database for Annotation, Visualization, and Integrated Discovery (DAVID) annotation tool (https://david.ncifcrf.gov/) to the identification of the main biological processes and pathways represented by DE mRNAs. A functional category was considered significant if it comprised at least five mRNAs and a score of *p* < 0.005 with Benjamini–Hochberg correction. The STRING algorithm (Search Tool for the Retrieval of Interacting Proteins database: https://string-b.org/cgi/input?sessionId=brAz10J8qufA&input_page_show_search=on), which establishes interactions among the gene encoded proteins based on the validated data retrieved from the literature, was used to construct networks.

### Reverse transcription quantitative real-time PCR (RT-qPCR)

The validation of the transcriptional expression of selected DE mRNAs was assayed by reverse transcription-quantitative real-time PCR (RT-qPCR) of the respective cDNAs. The expression level of each target mRNA was normalized to the housekeeping mRNA Hprt, which is commonly used as a reference. The Primer Blast (https://www.ncbi.nlm.nih.gov/tools/primer-blast/) web tool was used to select pairs of oligonucleotide primers spanning an intron/exon junction with an optimal melting temperature of 60 °C. The respective sequences were retrieved from the NCBI GenBank database (https://www.ncbi.nlm.nih.gov/). The forward (F) and reverse (R) primer sequences (presented in the 5′–3′ orientation) were as follows:

*Hprt* (NM_013556.2) F = 5' GCCCCAAAATGGTTAAGGTT 3' *R =* 5' CAAGGGCATATCCAACAACA 3', *Plcb2* (NM_177568.2) F = 5'TGGAGTTCCTGGATGTCACG3' *R =* 5'GCAGGAAGTGGTTGTCTGGA3', *Parvb* (NM_133167.3) F = 5' TCTTTCTTGGGCAAGTTGGG3' *R =* 5' CCATTGGAGAGTTGATGGCG3', *P2rx7* (NM_011027.4) F = 5'GCACGAATTATGGCACCGTC3' *R =* 5'TAACAGGCTCTTTCCGCTGG3', *Vegfc* (NM_009506.2) F = 5'GCTGATGTCTGTCCTGTACCC3' *R =* 5'ACTGTCCCCTGTCCTGGTAT3', *Id1* (NM_001369018.1) F = 5'CCTGAACGGCGAGATCAGTG3' *R =* 5'AAGTAAGGAAGGGGGACACC3'.

Gene expression was quantified using a StepOne Real-Time PCR System apparatus (Applied Biosystems). The analyses were performed using the cycle threshold (Ct) method, which allows for quantitative analysis of the expression of a factor using the formula 2 − ΔΔCt, in which ΔCt = Ct target gene − Ct of the housekeeping gene Hprt, and ΔΔCt = ΔCt sample − ΔCt. Experiments were performed in three independent replicates.

### Statistical analyses

Depending on the distribution of the variables in each comparison, statistical analyses were performed using the unpaired T or the Mann-Whitney U-tests, considering significant *p*-values < 0.05.

## Data Availability

The raw RNA-seq data from this work is available at Gene Expression Omnibus (GEO) (https://www.ncbi.nlm.nih.gov/geo/) under accession number PRJNA763914 available at (https://www.ncbi.nlm.nih.gov/bioproject/PRJNA763914).

## References

[1] Muñoz JJ, Zapata AG, Passos G (2019). Thymus Ontogeny and Development. Thymus Transcriptome and Cell Biology.

[2] Matsumoto M, Rodrigues PM, Sousa L, Tsuneyama K, Matsumoto M, Alves NL, Passos G (2019). The Ins and Outs of Thymic Epithelial Cell Differentiation and Function. Thymus Transcriptome and Cell Biology.

[3] Abramson J, Anderson G (2017). Thymic epithelial cells. Annu Rev Immunol.

[4] Takaba H, Takayanagi H (2017). The Mechanisms of T Cell Selection in the Thymus. Trends Immunol.

[5] Cosway EJ, Lucas B, James KD, Parnell SM, Carvalho-Gaspar M, White AJ, Tumanov AV, Jenkinson WE, Anderson G (2017). Redefining thymus medulla specialization for central tolerance. J Exp Med.

[6] Yoganathan K, Chen ELY, Singh J, Zúñiga-Pflücker JC, Passos G (2019). T-Cell Development: From T-Lineage Specification to Intrathymic Maturation. Thymus Transcriptome and Cell Biology.

[7] Derbinski J, Pinto S, Rösch S, Hexel K, Kyewski B (2008). Promiscuous gene expression patterns in single medullary thymic epithelial cells argue for a stochastic mechanism. Proc Natl Acad Sci U S A.

[8] Abramson J, Giraud M, Benoist C, Mathis D (2010). Aire’s Partners in the Molecular Control of Immunological Tolerance. Cell.

[9] Passos GA, Speck-Hernandez CA, Assis AF, Mendes-da-Cruz DA (2018). Update on Aire and thymic negative selection. Immunology.

[10] Passos GA, Genari AB, Assis AF, Monteleone- AC, Donadi EA, Oliveira EH, Duarte MJ, Machado MV, Tanaka PP, Mascarenhas R, Passos G (2019). The Thymus as a Mirror of the Body’s Gene Expression.

[11] St-Pierre C, Brochu S, Vanegas JR, Dumont-Lagacé M, Lemieux S, Perreault C. Transcriptome sequencing of neonatal thymic epithelial cells. Sci Rep. 2013;3. 10.1038/srep01860. 10.1038/srep01860 PMC3656389 23681267

[12] Sansom SN, Shikama-Dorn N, Zhanybekova S, Nusspaumer G, Macaulay IC, Deadman ME, Heger A, Ponting CP, Holländer GA (2014). Population and single-cell genomics reveal the Aire dependency, relief from Polycomb silencing, and distribution of self-antigen expression in thymic epithelia. Genome Res.

[13] Abramson J, Goldfarb Y (2016). AIRE: From promiscuous molecular partnerships to promiscuous gene expression. Eur J Immunol.

[14] Irla M, Passos G (2019). Thymic Crosstalk: An Overview of the Complex Cellular Interactions That Control the Establishment of T-Cell Tolerance. Thymus Transcriptome and Cell Biology.

[15] Giraud M, Yoshid H, Abramson J, Rahl PB, Young RA, Mathis D, Benoist C (2012). Aire unleashes stalled RNA polymerase to induce ectopic gene expression in thymic epithelial cells. Proc Natl Acad Sci U S A.

[16] Takaba H, Morishita Y, Tomofuji Y, Danks L, Nitta T, Komatsu N, Kodama T, Takayanagi H (2015). Fezf2 Orchestrates a Thymic Program of Self-Antigen Expression for Immune Tolerance. Cell.

[17] Pezzi N, Assis AF, Cotrim-Sousa LC, Lopes GS, Mosella MS, Lima DS, Bombonato-Prado KF, Passos GA (2016). Aire knockdown in medullary thymic epithelial cells affects Aire protein, deregulates cell adhesion genes and decreases thymocyte interaction. Mol Immunol.

[18] Speck-Hernandez CA, Assis AF, Felicio RF, Cotrim-Sousa L, Pezzi N, Lopes GS, et al. Aire disruption influences the medullary thymic epithelial cell transcriptome and interaction with thymocytes. Front Immunol. 2018;9. 10.3389/fimmu.2018.00964. 10.3389/fimmu.2018.00964 PMC5949327 29867946

[19] White A, Jenkinson E, Anderson G. Reaggregate thymus cultures. J Vis Exp. 2008;18. 10.3791/905. 10.3791/905 PMC3253565 19066499

[20] Tajima A, et al. Promoting 3-D Aggregation of FACS Purified Thymic Epithelial Cells with EAK 16-II/EAKIIH6 Self-assembling Hydrogel. J Vis Exp. 2016:112. 10.3791/54062 PMC4993281 27404995

[21] Mendes-Da-Cruz DA, Stimamiglio MA, Muñoz JJ, Alfaro D, Terra-Granado E, Garcia-Ceca J, Alonso-Colmenar LM, Savino W, Zapata AG (2012). Developing T-cell migration: Role of semaphorins and ephrins. FASEB J.

[22] Hubert FX, Kinkel SA, Crewther PE, Cannon PZ, Webster KE, Link M, Uibo R, O'Bryan MK, Meager A, Forehan SP, Smyth GK, Mittaz L, Antonarakis SE, Peterson P, Heath WR, Scott HS (2009). Aire-deficient C57BL/6 mice mimicking the common human 13-base pair deletion mutation present with only a mild autoimmune phenotype. J Immunol.

[23] Pinto S, Stark HJ, Martin I, Boukamp P, Kyewski B (2015). 3D Organotypic Co-Culture Model Supporting Medullary Thymic Epithelial Cell Proliferation, Differentiation and Promiscuous Gene Expression. J Vis Exp.

[24] Ucar A, Ucar O, Klug P, Matt S, Brunk F, Hofmann TG, Kyewski B (2014). Adult thymus contains foxN1- epithelial stem cells that are bipotent for medullary and cortical thymic epithelial lineages. Immunity.

[25] Sheridan JM, Keown A, Policheni A, Roesley SNA, Rivlin N, Kadouri N, Ritchie ME, Jain R, Abramson J, Heng TSP, Gray DHD (2017). Thymospheres Are Formed by Mesenchymal Cells with the Potential to Generate Adipocytes, but Not Epithelial Cells. Cell Rep.

[26] Silva CS, Pinto RD, Amorim S, Pires RA, Correia-Neves M, Reis RL, Alves NL, Martins A, Neves NM (2020). Fibronectin-Functionalized Fibrous Meshes as a Substrate to Support Cultures of Thymic Epithelial Cells. Biomacromolecules.

[27] Pérez AR, Berbert LR, Lepletier A, Revelli S, Bottasso O, Silva-Barbosa SD, et al. TNF-α is involved in the abnormal thymocyte migration during experimental trypanosoma cruzi infection and favors the export of immature cells. PLoS One. 2012;7(3). 10.1371/journal.pone.0034360. 10.1371/journal.pone.0034360 PMC3312912 22461911

[28] Anderson G, Jenkinson EJ (2007). Fetal Thymus Organ Culture. Cold Spring Harb Protoc.

[29] Kyewski B, Klein L (2006). A central role for central tolerance. Annu Rev Immunol.

[30] Hogquist KA, Baldwin TA, Jameson SC (2005). Central tolerance: Learning self-control in the thymus. Nat Rev Immunol.

[31] Brunk F, Michel C, Holland-Letz T, Slynko A, Kopp-Schneider A, Kyewski B, Pinto S (2017). Dissecting and modeling the emergent murine TEC compartment during ontogeny. Eur J Immunol.

[32] Sun L, Li H, Luo H, Zhao Y. Thymic epithelial cell development and its dysfunction in human diseases. Biomed Res Int. 2014;2014. 10.1155/2014/206929. 10.1155/2014/206929 PMC3929497 24672784

[33] St-Pierre C, Trofimov A, Brochu S, Lemieux S, Perreault C (2015). Differential Features of AIRE-Induced and AIRE-Independent Promiscuous Gene Expression in Thymic Epithelial Cells. J Immunol.

[34] Da R, Bisaggio C, Nihei OK, Persechini PM, Fundação WS, Cruz O (2017). Characterization of P2 receptors in thymic epithelial cells. Cell Mol Biol.

[35] Nihei OK, Campos de Carvalho AC, Spray DC, Savino W, Alves LA (2003). A novel form of cellular communication among thymic epithelial cells: Intercellular calcium wave propagation. Am J Phys Cell Phys.

[36] Hirokawa K, Utsuyama M, Moriizumi E, Handa S (1986). Analysis of the thymic microenvironment by monoclonal antibodies with special reference to thymic nurse cells. Thymus.

[37] Mizuochi T, Kasai M, Kokuho T, Kakiuchi T, Hirokawa K (1992). Medullary but not cortical thymic epithelial cells present soluble antigens to helper T cells. J Exp Med.

